# Enhancing immunity prevents virus‐induced T‐cell‐mediated immunopathology in B cell‐deficient mice

**DOI:** 10.1002/eji.201847962

**Published:** 2019-03-01

**Authors:** Tobias Straub, Hanspeter Pircher

**Affiliations:** ^1^ Institute for Immunology, Medical Center ‐ University of Freiburg Faculty of Medicine, University of Freiburg Germany

**Keywords:** antiviral T cells, disease, immunodeficiency, immunopathology, vaccination

## Abstract

Hyper‐activated or deviated immune responses can result in immunopathological diseases. Paradoxically, immunodeficiency represents a frequent cause of such immune‐mediated pathologies. Immunopathological manifestations are commonly treated by immunosuppression, but in situations in which immunodeficiency is the basis of disease development, enhancing immunity may represent an alternative treatment option. Here, we tested this counterintuitive concept in a preclinical model using infection of mice with lymphocytic choriomeningitis virus (LCMV). Firstly, we demonstrate that infection of B‐cell‐deficient (B^−/−^) but not of wild‐type (WT) mice with the LCMV strain Docile induced a rapid and fatal CD8^+^ T‐cell‐mediated immunopathological disease. Similar to WT mice, LCMV‐infected B^−/−^ mice generated a potent, functional LCMV‐specific CD8^+^ T‐cell response but exhibited prolonged viral antigen presentation and increased vascular leakage in liver and lungs. Secondly, we were able to prevent this virus‐induced immunopathology in B^−/−^ mice by active or passive T‐cell immunizations or by treatment with LCMV‐specific virus neutralizing or non‐neutralizing monoclonal antibodies (mAb). Thus, boosting antiviral immunity did not aggravate immunopathology in this model, but prevented it by decreasing the formation of target structures for damage‐causing CD8^+^ T cells.

## Introduction

Hyper‐activated or deviated immune responses can result in immunopathological diseases. Paradoxically, immunodeficiency is a frequent cause of such immune‐mediated pathologies. This is illustrated for example in patients with common variable immunodeficiency (CVID) who often present with immunopathological disorders 1. Also in chronic viral hepatitis, a partially defective T cell response contributes to liver disease pathogenesis 2, 3. There, immune dysregulation as well as inefficient pathogen control presumably lead to continuous activation of immune cells that cause devastating immunopathological damage. Immune‐mediated diseases are usually treated by immunosuppression. Although it is counterintuitive, improving immunity may represent an alternative treatment option in situations where immunodeficiency is the underlying cause for the induction of immunopathological reactions. In the present study, we tested this concept by using B cell‐deficient mice as an immunodeficiency model and LCMV as an infectious trigger. We demonstrate that B cell‐deficiency leads to a rapid and fatal CD8^+^ T cell‐mediated immunopathology after infection with the LCMV strain Docile. Importantly, we were able to prevent this immunopathology by strengthening antiviral immunity.

## Results and discussion

### Fatal CD8^+^ T cell‐mediated immunopathology in B cell‐deficient mice after LCMV Docile infection

**Figure 1:**
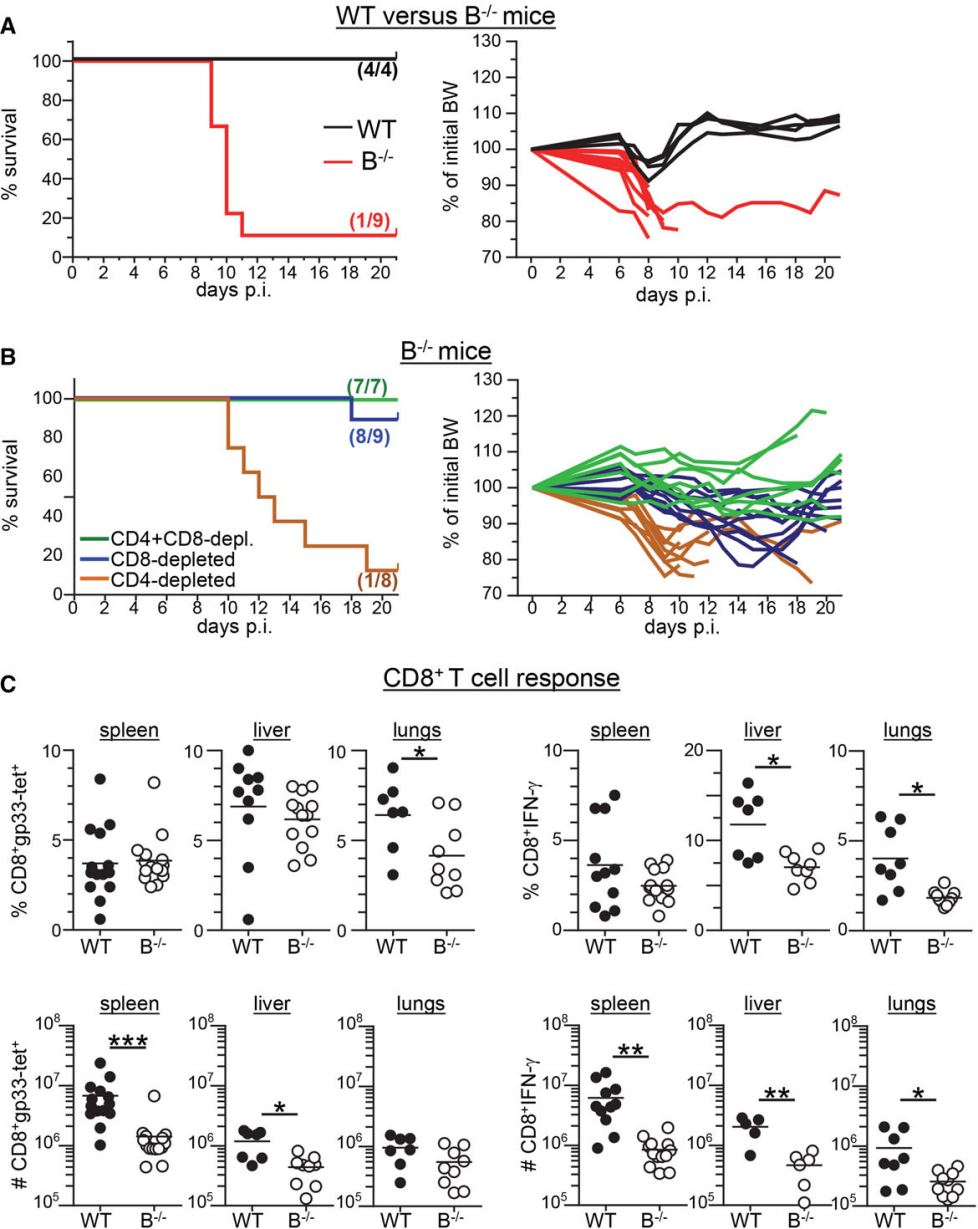
Fatal CD8^+^ T cell‐mediated immunopathology in B^−/−^ mice after LCMV Docile infection. (A) WT (black, *n* = 4) and B^−/−^ mice (red, *n* = 9) were infected with 200 pfu LCMV Docile. Survival and body weight (BW) were monitored for the indicated time period. Data shown are pooled from three (B^−/−^) or one (WT) experiment with 2–4 mice per group. (B) B^−/−^ mice were treated with anti‐CD8 Ab (blue, *n* = 9), anti‐CD4 Ab (brown, *n* = 8) or anti‐CD4 + anti‐CD8 Ab (green, *n* = 7) before infection with 200 pfu LCMV Docile. Survival and BW were monitored for the indicated time period. The number of surviving mice of total infected mice (n/n) for each setting is indicated in the corresponding graph. Data shown are pooled from two to three independent experiments with three to four mice per group. (C) B6 WT (filled circles) and B^−/−^ mice (open circles) were infected with 200 pfu LCMV Docile. At day 9 p.i., the LCMV‐specific CD8^+^ T cell response was determined by MHC class I tetramer (D^b^/gp33) staining and by staining for intracellular IFN‐γ after restimulation with LCMV gp33 peptide. Values of gp33 tet^+^CD8^+^ T cells (left) and of IFN‐γ^+^CD8^+^ T cells (right) in percent (upper row) and absolute numbers (#; lower row) of spleen cells and of leukocytes isolated from livers and lungs are depicted. Symbols represent values from individual mice (*n* = 11–15 (spleen), 5–13 (liver), 7–10 (lungs)), horizontal lines indicate means; data are pooled from two to six independent experiments with 2–3 mic per group. *p < 0.05, **p < 0.01, ***p < 0.001; unpaired *t*‐test with Welch correction or Mann–Whitney test.

Inoculation of wild‐type (WT) C57BL/6 (B6) mice with a low dose (200 pfu) of LCMV strain Docile induces an acute infection with virus elimination within 2–3 weeks post infection (p.i.) 4. Importantly, WT mice did not show clinical symptoms after this infection and developed only a transient, small (∼5%) decrease in body weight (BW). In striking contrast, B cell‐deficient J_H_T mice (henceforth referred to as B^−/−^ mice) rapidly lost weight and almost all of them died or had to be euthanized due to their moribund conditions at day 8–10 p.i. (Fig. 1A). Since LCMV is a non‐cytopathic virus, the disease in B^−/−^ mice was likely to be caused by the induced immune response. Indeed, antibody (Ab)‐mediated depletion of CD8^+^ T cells in B^−/−^ mice prevented death in 8 out of 9 mice as well as significant weight loss. Depletion of CD4^+^ T cells alone had no beneficial effect on survival but when combined with anti‐CD8 Ab treatment, it decreased BW loss in comparison to the anti‐CD8 Ab‐treated group (Fig. 1B). Of note, T cell‐depleted B^−/−^ mice exhibited high viral titers at the end of the observation period (day 21 p.i.) (Supporting Information Fig. 1). This further supports the notion that T cells but not the virus itself were responsible for the observed lethal pathology in B^−/−^ mice. In sum, these data show that the LCMV Docile infection induced a rapid and fatal CD8^+^ T cell‐mediated immunopathological disease in mice lacking B cells.

### Prolonged antigen presentation and vascular leakage in B^−/−^ mice after LCMV Docile infection

**Figure 2:**
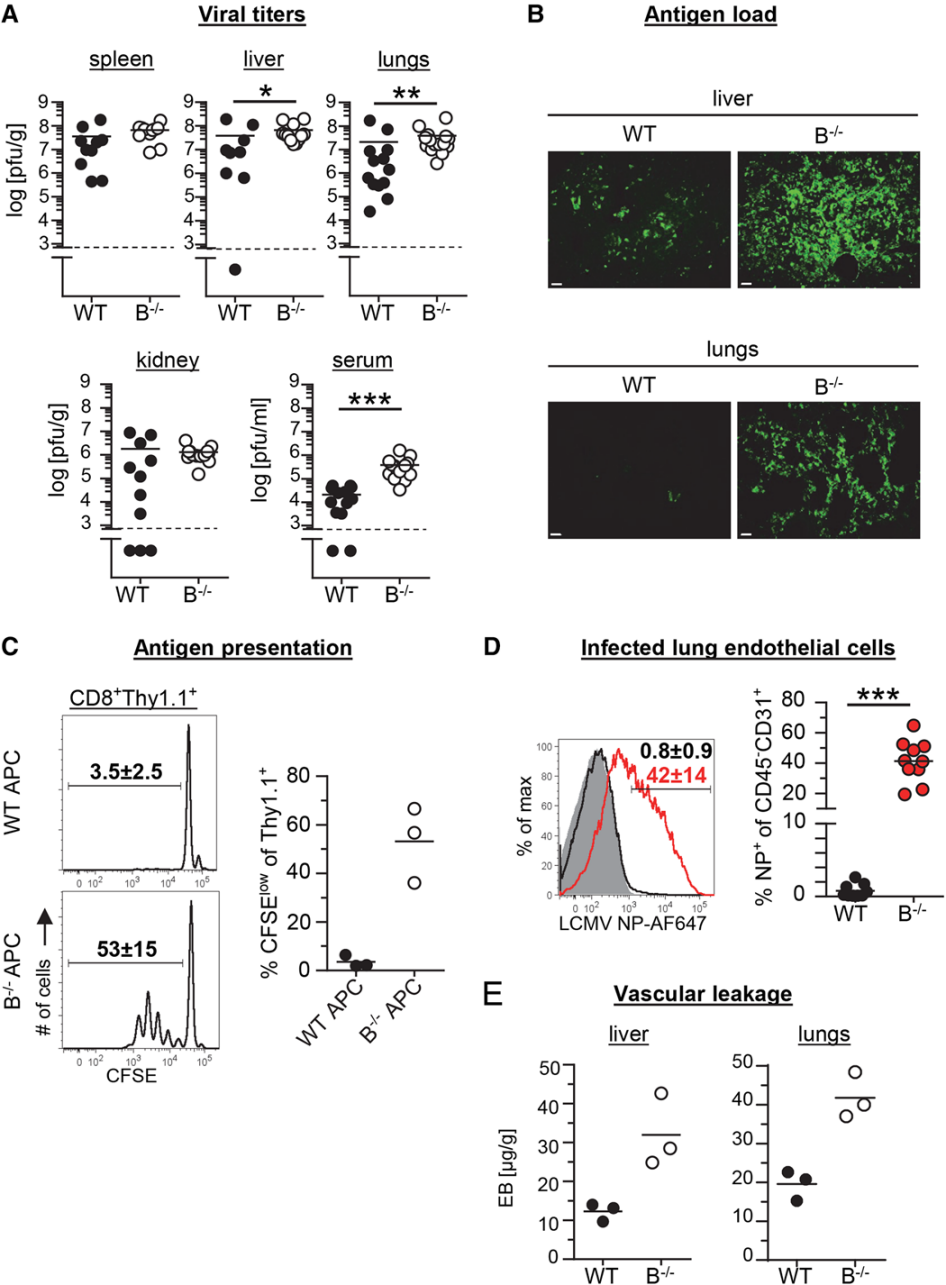
Viral load, endothelial cell infection and vascular leakage in LCMV Docile‐infected mice. WT (solid circles) and B^−/−^ mice (open circles) were infected with 200 pfu LCMV Docile and analysed at day 9 p.i. (A) Viral titers in the indicated organs and in serum. Symbols represent values from individual mice (*n* = 9–15); data are pooled from five to six independent experiments with two to three mice per group. (B) Frozen sections from liver and lungs were stained with an anti‐LCMV immune serum and analyzed by immunofluorescence microscopy. Exemplary images of two independent experiments (*n* = 6–7) performed with three to four mice per group are shown. Original magnification: 10x. Scale bar: 50 μm. (C) CFSE‐labeled Thy1.1^+^ P14 TCR tg CD8^+^ T cells were stimulated in vitro with lung APC (leukocytes depleted of B and T cells) from infected mice and analyzed for dye dilution 3 days later. On the left, representative histograms are shown; on the right, data from one representative experiment (*n* = 3) of two independent experiments with three to four mice per group are depicted. (D) Intracellular staining of lung endothelial cells (CD45^−^CD31^+^) with LCMV NP‐specific mAb VL4. Representative histogram (grey: uninfected control) and pooled data from three independent experiments (*n* = 9–10) with three to four mice per group are depicted. Symbols represent values from individual mice, numbers in the histogram indicate mean percentages ± s.d. (E) Concentrations of Evans Blue (EB) retained in perfused liver and lungs of LCMV‐infected mice (day 9 p.i.) 15 min after i.v. injection. Data from one representative experiment (*n* = 3) of two independent experiments with three to four mice per group are depicted. *p < 0.05, **p < 0.01, ***p < 0.001; Mann–Whitney‐test.

To characterize the disease in LCMV‐infected B^−/−^ mice, we first measured the LCMV‐specific CD8^+^ T cell response in spleen, liver and lungs by MHC class I tetramer (gp33‐tet) and intracellular IFN‐γ staining at day 9 p.i. Functional LCMV‐specific CD8^+^ T cells were detected in B^−/−^ mice but their absolute numbers were somewhat lower than in WT mice (Fig. 1C). In addition, we observed decreased KLRG1 expression in CD8^+^ T cell from LCMV‐infected B^−/−^ mice when compared to WT mice (Supporting Information Fig. 2). Next, we assessed the amount of infectious virus in various organs by focus forming assay (Fig. 2A). At day 9 p.i. infectious viral particles were slightly more numerous in spleen, liver, lungs and kidneys of B^−/−^ mice when compared to WT mice; in serum, B^−/−^ mice had ∼10‐fold higher levels of virus than WT mice. Viral protein as determined by immunohistology of liver and lungs was also more abundant in B^−/−^ mice than in WT mice (Fig. 2B). In addition, antigen presenting cells (APC) isolated ex vivo from lungs of LCMV‐infected B^−/−^ but not those isolated from infected WT mice were able to stimulate LCMV‐specific P14 T cell receptor (TCR) transgenic (tg) CD8^+^ T cells (Fig. 2C).

In the LCMV clone 13 chronic infection model, infected vascular endothelial cells have been shown to serve as target cells for LCMV‐specific cytotoxic T lymphocytes (CTL). In this model, PD‐1‐deficiency led to enhanced killing of infected endothelial cells by virus‐specific CTL followed by systemic vascular leakage and collapse of the circulatory system 5. Interestingly, most of the lung endothelial cells (CD45^−^CD31^+^) from LCMV‐infected B^−/−^ mice were infected with LCMV as deduced from the strong intracellular staining with a LCMV nucleoprotein (NP)‐specific mAb (Fig. 2D). In contrast, LCMV NP could not be detected in lung endothelial cells from LCMV‐infected WT mice. Finally, levels of Evans blue (EB) dye in liver and lungs of LCMV‐infected B^−/−^ mice extracted 15 min. after i.v. injection were considerably increased in comparison to WT mice, indicating enhanced vascular leakage (Fig. 2E). Thus, the immunopathological features observed in LCMV Docile‐infected B^−/−^ mice were similar to those described for PD‐1‐deficient mice 5 or for particular mouse strains 6, 7 after LCMV clone 13 infection.

Our data show that B^−/−^ mice were unable to contain the LCMV Docile infection in contrast to WT mice, resulting in prolonged LCMV antigen presentation to CD8^+^ T cells. B cell deficiency may impact LCMV control in several ways. Firstly, B^−/−^ mice are unable to produce antibodies and rapidly generated, non‐neutralizing LCMV‐specific Ab have been shown to speed up virus elimination 4, 8, 9. Secondly, B cell‐deficient mice lack a normal splenic marginal zone 10 which leads to faster spread of virus to peripheral organs 11. Thirdly, the lack of the splenic marginal zone also results in an impaired type I interferon production 12.

B^−/−^ mice do not develop immunopathological disease after infection with the LCMV strains Armstrong and WE 13, 14. These viral strains exhibit lower replication speed when compared to the Docile strain, indicating that high antigen load and/or fast spreading of the virus to peripheral organs was required for the development of immunopathological disease. However, infection of WT mice with a high dose (2 × 10^6^ pfu) of LCMV Docile which results in high viral titers does not trigger fatal disease (15 and Supporting Information Fig. 3). Furthermore, B cell receptor‐transgenic MD4 mice specific for hen egg lysozyme which are also unable to contain a LCMV Docile infection did not develop disease (Supporting Information Fig. 4). Thus, lack of B cells appeared to be crucial for the morbidity after LCMV Docile infection. At an early time point (day 4) of infection, B^−/−^ mice exhibited significantly lower viral titers in spleen but increased titers in liver and lungs when compared to WT mice (Supporting Information Fig. 5). Hence, it is possible that due to initially low viral load in spleen, exhaustive T cell differentiation is impaired or delayed in B^−/−^ mice. Nonetheless, decreased PD‐1 expression on CD8^+^ T cells or decreased PD‐L1 expression on lung endothelial cells was not observed in infected B^−/−^ mice (Supporting Information Fig. 6). This argues against a failure of PD‐1‐PD‐L1‐mediated inhibition of CD8^+^ T cells as underlying cause of the observed pathology.

### Prevention of LCMV‐induced immunopathology in B^−/−^ mice by enhancing immunity

**Figure 3:**
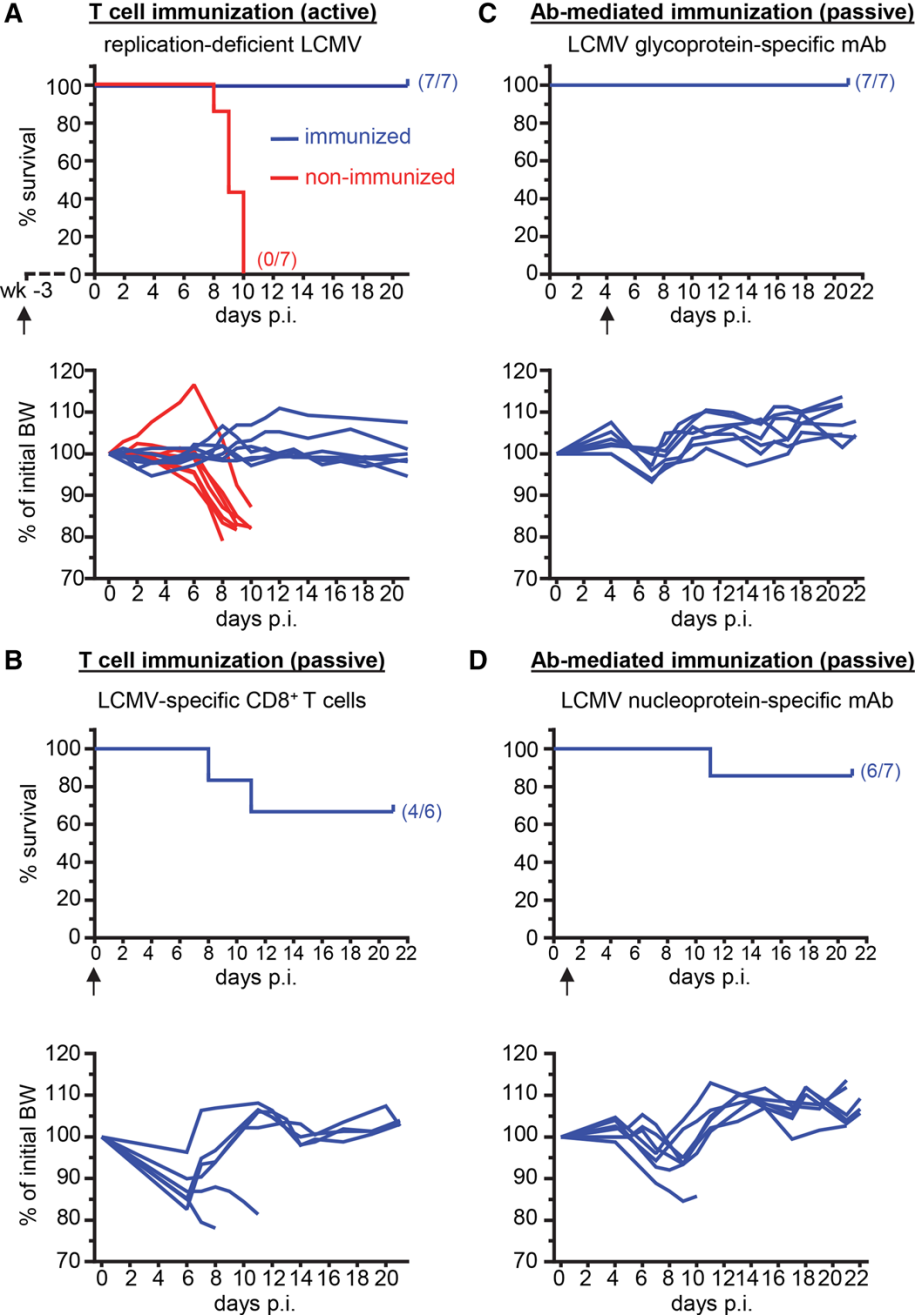
Prevention of LCMV‐induced immunopathology in B^−/−^ mice by enhancing immunity. (A) B^−/−^ (blue, *n* = 7) mice were immunized with replication‐deficient rLCMV/WEGPΔGlc6,9 (8 × 10^4^ pfu) 3 weeks prior to challenge with 200 pfu LCMV Docile. Non‐immunized B^−/−^ mice (red, *n* = 7) were included as a controls. (B) B^−/−^ mice (*n* = 6) were adoptively transferred (i.v.) with 5 × 10^5^ P14 TCR tg CD8^+^ T cells at the day of infection with 200 pfu LCMV Docile. (C) LCMV Docile‐infected B^−/−^ mice (*n* = 7) were treated once with neutralizing LCMV GP‐specific mAb KL25 (1 mg) at day 4 p.i. (D) LCMV Docile‐infected B^−/−^ mice (*n* = 7) were treated once with non‐neutralizing LCMV NP‐specific mAb VL4 (500 μg) at day 1 p.i. (A‐D) Survival and body weight (BW) were monitored for immunized (blue) and non‐immunized (red) mice for the indicated time periods after infection. The number of surviving mice of total infected mice (n/n) for each setting is indicated in the corresponding graph. For each setting, data are pooled from two independent experiments with three to four mice per group. Arrows below the time axis indicate the time point of immunization.

Our data so far indicated that B^−/−^ mice rapidly develop a fatal CD8^+^ T cell‐mediated immunopathological disease after LCMV Docile infection due to concurrent presence of functional LCMV‐specific CD8^+^ T cells and target cells expressing LCMV antigens. We thus hypothesized that enhancing LCMV‐specific CD8^+^ T cell immunity may prevent CD8^+^ T cell‐mediated immunopathology by reducing the number of infected cells in the body. To test this idea, we vaccinated B^−/−^ mice with a replication‐deficient LCMV strain (rLCMV/WEGPΔGlc6,9) three weeks prior to challenge with LCMV Docile. Indeed, all (7/7) vaccinated B^−/−^ mice survived the LCMV Docile infection whereas all non‐vaccinated mice (7/7) died or had to be euthanized due to their moribund condition (Fig. 3A). Moreover, increasing the precursor frequency of LCMV‐specific CD8^+^ T cells in B^−/−^ mice by adoptive transfer of LCMV‐specific P14 TCR tg CD8^+^ T cells (5 × 10^5^) from uninfected donor mice also had a protective effect in 4 out 6 B^−/−^ mice (Fig. 3B).

The absence of a LCMV‐specific Ab response in B^−/−^ mice likely contributes to the impaired viral control in these mice. Therefore, we also examined whether passive immunizations with LCMV‐specific Ab were able to mitigate disease development. We first tested the virus‐neutralizing LCMV glycoprotein (GP)‐specific mAb KL25 that was injected 4 days after infection to allow for initial virus replication. The experiments revealed that KL25 mAb treatment protected all (7/7) B^−/−^ mice from death after LCMV Docile infection (Fig. 3C). In addition, we also used a non‐neutralizing LCMV NP‐specific mAb (VL4, treatment day 1 p.i.), since we had shown previously that also non‐neutralizing LCMV NP‐specific Ab were able to enhance CD8^+^ T cell‐mediated viral control in WT mice 4. Strikingly, treatment of LCMV Docile‐infected B^−/−^ mice with VL4 mAb at day 1 p.i. protected 6 out of 7 mice from immunopathology (Fig. 3D). Vaccination or Ab‐treatment of LCMV‐infected B^−/−^ mice led to strongly reduced viral load at the end of the observation period (Supporting Information Fig. 7). Moreover, P14 TCR tg T cell transfer or Ab‐treatment prevented high viral load and infection of lung endothelial cells already at day 9 p.i., the time point of disease development in infected non‐treated B^−/−^ mice (Supporting Information Fig. 8). These findings fit well to our concept that enhancing anti‐LCMV immunity in B^−/−^ mice decreases target cell structures for CD8^+^ T cells and thereby prevents LCMV‐induced immunopathology.

### Concluding remarks

The growing awareness that immunodeficiency can be the underlying cause of immunopathology requires a rethinking of the therapeutic strategies commonly used to treat such diseases. Here, we provide a proof‐of‐concept that immunopathology that is due to impaired immunity in the context of a viral infection, can be prevented by enhancing immunity. Immunopathology in B^−/−^ mice after LCMV Docile infection is caused by a functional antiviral CTL response and the concurrent presence of a high number of infected cells.

When we enhanced immunity by active or passive immunization strategies, this did not aggravate the immunopathological processes. In contrast, it reduced antigen load and prevented immune‐mediated disease. Interestingly, treatment of LCMV Docile‐infected B^−/−^ mice with non‐neutralizing LCMV nucleoprotein (NP)‐specific Ab was also found to be effective. This finding is well in line with our previous data demonstrating the LCMV NP‐specific Ab are capable of speeding up LCMV elimination in WT mice independently of complement and Fcγ receptors 4. In conclusion, the present study encourages further attempts to evaluate the concept of enhancing immunity to prevent immunopathology also in other settings.

## Materials and methods

### Mice

C57BL/6NRj mice were purchased from Janvier (Le Genest St‐Isle, France). B cell‐deficient J_H_T mice (B6.129P2‐*Igh‐J^tm1Cgn^*/J) 16 and their transgene‐negative littermates that were used as WT controls in some experiments and P14 TCR‐tg mice (B6;D2‐Tg(TcrLCMV)318Sdz/JDvsJ) 17 were bred locally. Mice were housed under specific pathogen‐free conditions. Animal care and use was approved by the Regierungspräsidium Freiburg. All experiments were performed according to the german law for animal protection.

### Viral infections

Mice were infected intravenously (i.v.) with 200 pfu LCMV strain Docile unless stated otherwise. LCMV was propagated on Madin Darby canine kidney (MDCK) cells. Viral titers were determined by focus‐forming assay 18. For active immunization, mice were injected i.v. with 8 × 10^4^ pfu of replication‐deficient rLCMV/WEGPΔGlc6,9 carrying a modified glycoprotein 19, 20 three weeks before challenge infection with LCMV Docile.

### Antibody treatments

T cell depletion in vivo was performed by intraperitoneal (i.p.) injection of 200 μg purified anti‐CD4 (clone YTS191, BioXcell) and/or anti‐CD8 (clone YTS169, BioXcell) mAb at day 3 and day 1 before LCMV infection. For passive immunization, mice received 1 mg purified LCMV GP‐specific mAb KL25 21 (own production) at day 4 p.i. or 500 μg purified LCMV NP‐specific mAb (clone VL4, BioXcell) at day 1 p.i. via the intraperitoneal route.

### Flow cytometry

Flow cytometry was performed according to the guidelines as published by Cossarizza et al. 22. Single‐cell suspensions of lymphocytes were prepared from spleens by meshing through a metal strainer with a syringe plunger. Lymphocytes from liver were isolated by digestion with Collagenase D (1 mg ml^−1^, Roche) and DNaseI (0.1 mg ml^−1^, Sigma) followed by meshing the tissue through cell strainers (Greiner) and purification using Percoll gradient (Sigma). Lungs were digested using Collagenase II (140 U ml^−1^_,_ Roche) and DNaseI (0.1 mg ml^−1^) and meshed through a cell strainer. The following mAbs were used: anti‐CD8a (clone 53–6.7, AlexaFluor488, BioLegend), anti‐CD31 (clone ER‐MP12, biotinylated, MCA), anti‐PD‐L1 (clone 10F.9G2, PE, BioLegend), anti‐KLRG1 (2F1, PerCP‐Cy5.5, BioLegend), anti‐PD1 (29F.1A12, APC, BioLegend) and anti‐CD45 (clone 30‐F11, AlexaFluor700, BioLegend). LCMV‐specific CD8^+^ T cells were quantified by MHC class I tetramer staining using PE‐labeled D^b^gp33‐tetramers (produced in‐house). IFN‐γ‐production of CD8^+^ T cells was determined by intracellular (Cytofix/Cytoperm, BD Bioscience) IFNγ‐staining (clone XMG1.2, PE, eBioscience) after restimulation of 10^6^ lymphocytes with 10^−7^ M LCMV gp33 peptide in the presence of 15 μg ml^−1^ Brefeldin A (Sigma) for 4–5 h. LCMV infection of CD31^+^ endothelial cells was analyzed by intracellular staining (Cytofix/Cytoperm, BD Bioscience) using AlexaFluor647‐labeled (Thermo Fisher) LCMV NP‐specific mAb VL4 (BioXcell). Zombie NIR dye (BioLegend) or DAPI (Sigma) was used for dead cell exclusion. Staining was performed for at least 20 min at 4°C. Samples were fixed in 4% formaldehyde and measured on a LSR Fortessa or Canto II cytometer (both BD Biosciences) and data were analyzed with FlowJo software 8.8.7 (Tree Star). Gating strategies are shown in Supporting Information Fig. 9.

### In vitro P14 T cell stimulation assay

Leukocytes from collagenase‐ and DNase‐digested lungs (see above) were isolated by pressing through cell strainers. Suspensions were depleted of B and T cells by staining with PE‐conjugated anti‐Thy1.2 (clone 53‐2.1, eBioscience) and anti‐B220 (clone RA3‐6B2, eBioscience) Ab, followed by magnetic separation with anti‐PE MicroBeads (Miltenyi) according to the manufacturer's instructions. The resulting cells (>80% CD45^+^) were co‐cultured at a 1:1 ratio with 1 × 10^5^ CFSE‐labeled enriched (mouse CD3 T cell isolation kit, BioLegend) P14 CD8^+^ T cells in 96‐well cell culture plates (Thermo Fisher) for 3 days. Afterwards, cell division of P14 T cells identified by the allelic Thy1.1 marker was analyzed by CFSE dye dilution.

### Immunohistology

Livers and lungs were fixed in 4% formaldehyde, immersed for 3 hours in 15% sucrose, then overnight in 30% sucrose and finally embedded in Tissue‐Tek O.C.T. compound (SAKURA), snap‐frozen in liquid nitrogen and stored at −80°C. 8 μm sections were prepared by a cryostat (Leica CM1850), put on superfrost slides (R. Langenbrinck) and blocked with PBS containing 5% mouse serum and 5% goat serum. Staining was performed for 1h at room temperature with a rabbit‐anti‐LCMV serum (own production) followed by AlexaFluor488‐conjugated goat‐anti‐rabbit IgG Ab (Thermo Fisher). Images were acquired using the Axioplan2 imaging microscope with AxioCam MRm and Plan‐NEOFLUAR 10x/0.30 objective and analyzed by AxioVision software (all from Zeiss).

### Analysis of vascular permeability

Vascular permeability was assessed by Evans blue (EB) extravasation assay as described previously 5, 6. In brief, mice were injected i.v. with 200 μL PBS containing 0.5% EB dye. After 15 min, mice were lethally anesthetized and transcardially perfused with 10 ml PBS. Liver and lungs were excised, weighed and incubated for 24 hours in 2 ml formamide at 56°C. The amount of extracted EB was determined by photometrical analysis at 620 nm using a standard curve.

### Statistics

Statistical differences between two groups were determined using unpaired two‐tailed *t*‐test with Welch‐correction or Mann‐Whitney Test depending on whether data demonstrated Gaussian distribution or not. GraphPad InStat 3 software was used for determination of p‐values. Differences at values of *p < 0.05, **p < 0.01 and ***p < 0.001 were considered to be significant.

## Conflict of interest

The authors declare no financial or commercial conflict of interest.

**Abbreviations**
Ab: antibody
B6: C57BL/6
APC: antigen presenting cells
BW: body weight
CTL: cytotoxic T lymphocytes
EB: Evans blue
GP: glycoprotein
i.v.: intravenously
LCMV: lymphocytic choriomeningitis virus
(m)Ab: (monoclonal) antibody
NP: nucleoprotein
PBS: phosphate buffered saline
p.i.: post infection
pfu: plaque forming unit
TCR: T cell receptor
tg: transgenic
WT: wild‐type

## Supporting information

Supporting Information Fig. 1: CD8+ T cell‐depleted B‐/‐ mice were infected with 200 pfu LCMV Docile. At day 21 p.i. viral titers in spleen, liver and lungs were determined by focus‐forming assay. Symbols represent data from individual mice; data from one experiment are shown (n = 3). The horizontal lines indicate the means, the dashed lines indicate the detection limit.
Supporting Information Fig. 2: WT and B‐/‐ mice were infected with 200 pfu LCMV Docile. At day 9 p.i. (A) percentages of KLRG1+ cells among CD8+ T cell and (B) percentages of KLRG1+ cells of gp33‐tet+CD8+ T cells were determined. Symbols represent data from individual mice; data shown are pooled from (A) five (spleen, liver; n = 12) or three (lung; n = 8‐9) independent experiments with 2‐3 mice per group or (B) two independent experiments (n = 5‐6) with 2‐3 mice per group. *p < 0.05, unpaired t‐test with Welch correction.
Supporting Information Fig. 3: WT mice were infected with 2x106 pfu LCMV Docile. (A) Survival and body weight (BW) were monitored for the indicated time period. (B) Viral titers were determined in the indicated organs at day 21 p.i. Symbols represent data from individual mice; horizontal lines indicate means, dashed lines indicate the detection limit. Pooled data (n = 6) from three independent experiments with 1‐3 mice per experiment.
Supporting Information Fig. 4: (A, B) WT and MD4 BCR‐transgenic mice specific for hen egg lysozyme (HEL) (Hartley et al., Nature 1991, 353:765‐9) were infected with 200 pfu LCMV Docile. (A) Survival and body weight (BW) were monitored for the indicated time period. (B) Viral titers were determined in the indicated organs at day 9 p.i. Symbols represent data from individual mice; horizontal lines indicate means, dashed lines indicate the detection limit. Data shown are pooled from (A) one (WT, n = 4) or two (MD4, n = 6) independent experiments with 3‐4 mice per experiment or (B) 5‐6 independent experiments (n = 12‐13) with 2‐3 mice per group. *p < 0.05, **p < 0.01; Mann‐Whitney test.
Supporting Information Fig. 5: B‐/‐ mice were infected with 200 pfu LCMV Docile. At day 4 p.i. (A) viral titers in spleen, liver and lungs were determined. Symbols represent data from individual mice; horizontal lines indicate means, dashed lines indicate the detection limit. Data are pooled from 2‐3 independent experiments (n = 7) with 2‐4 mice per group; (B) splenic sections were stained with rabbit anti‐LCMV immune serum and biotinylated anti‐F4/80 mAb (clone A3‐1, AbD Serotec), followed by streptavidin‐ AlexaFluor555 (red) and AlexaFluor488‐labeled goat anti‐rabbit IgG (green, both invitrogen). Exemplary images of two independent experiments are shown. *p < 0.05, **p < 0.01, Mann‐Whitney test.
Supporting Information Fig. 6: WT and B‐/‐ mice were infected with 200 pfu LCMV Docile. At day 9 p.i. (A) expression level (geometric mean fluorescence intensity, gMFI) of PD‐L1 on CD45‐CD31+ lung endothelial cells and (B) frequency of PD1+ of CD8+ T cells as well as (C) the expression level (gMFI) of PD1 on gp33‐tet+CD8+ T cells was determined. (A) A representative histogram of one experiment with 3 mice per group (black: WT; red: B‐/‐; grey: uninfected control; numbers to the right indicate gMFI ± s.d.) and (B, C) pooled data from (B) five (spleen, liver; n = 12) or three (lung; n = 8‐9) independent experiments with 2‐3 mice per group or (C) two independent experiments (n = 5‐6) with 2‐3 mice per group are shown. Symbols represent data from individual mice, horizontal bars indicate the means. *p < 0.05, unpaired t‐test with Welch correction.
Supporting Information Fig. 7: (A) B‐/‐ mice were vaccinated (vacc.) with replication‐deficient rLCMV/WEGPΔGlc6,9 (8 x 104 pfu) three weeks prior to challenge with 200 pfu LCMV Docile. Nonvaccinated B‐/‐ mice were included as a control (ctrl). Viral titers were determined by focus‐forming assay at day 4 p.i. (B) B‐/‐ mice were adoptively transferred (i.v.) with 5 x 105 P14 TCR tg CD8+ T cells at the day of infection with 200 pfu LCMV Docile. Viral titers were determined at day 21 p.i. (C) LCMV Docile‐infected B‐/‐ mice were treated once with neutralizing LCMV GP‐specific mAb KL25 (1 mg) at day 4 p.i. At day 21, viral load was determined by qPCR. (D) LCMV Docile‐infected B‐/‐ mice were treated once with nonneutralizing LCMV NP‐specific mAb VL4 (0.5 mg) at day 1 p.i. Viral titers were determined at day 21 p.i. using focus‐forming assay. (A‐D) Symbols represent values from individual mice, horizontal lines indicate means, dashed lines indicate the detection limit; data are pooled from two to three independent experiments; uninfected and infected controls in (C) are derived from one experiment (n = 1).
Supporting Information Fig. 8: B‐/‐ mice were infected with 200 pfu LCMV Docile and treated with (A) 5x105 P14 T cells (day 0 p.i.), (B) 1 mg mAb KL25 (day 4 p.i.) or (C) 500 μg mAb VL4 (day 1 p.i.). At day 9 p.i. (A, C, E) viral load in spleen, liver and lungs and (B, D, F) frequency of LCMV NP+ lung endothelial cells (CD45‐CD31+) were determined. Pooled data and representative histograms from three (A, C, E) or two (B, D, F) independent experiments are shown (n = 8‐10 or n = 5‐6, respectively) with 2‐4 mice per experiment. Data for infected and uninfected controls in (C) are derived from one experiment (n = 3). Viral loads from untreated controls are shown in Fig. 2A. (A, C, E) Symbols represent data from individual mice; horizontal lines indicate means, dashed lines indicate detection limit. (C, D, F) Numbers in histograms indicate percentage of LCMV NP+ cell ± s.d.
Supporting Information Fig. 9: (A) Gating strategy used in Figure 1C, (B) Gating Strategy used in Figure 2D and E
