# Supplementary material for: Enhancing immunity prevents virus‐induced T‐cell‐mediated immunopathology in B cell‐deficient mice
Source: Eur J Immunol. 2019 Mar 1;49(5):782–9. doi: 10.1002/eji.201847962 (PMC6593698; doi:10.1002/eji.201847962)
Supplement: Supplementary file 1 — Supporting Information Fig. 1: CD8+ T cell‐depleted B‐/‐ mice were infected with 200 pfu LCMV Docile. At day 21 p.i. viral titers in spleen, liver and lungs were determined by focus‐forming assay. Symbols represent data from individual mice; data from one experiment are shown (n = 3). The horizontal lines indicate the means, the dashed lines indicate the detection limit. Supporting Information Fig. 2: WT and B‐/‐ mice were infected with 200 pfu LCMV Docile. At day 9 p.i. (A) percentages of KLRG1+ cells among CD8+ T cell and (B) percentages of KLRG1+ cells of gp33‐tet+CD8+ T cells were determined. Symbols represent data from individual mice; data shown are pooled from (A) five (spleen, liver; n = 12) or three (lung; n = 8‐9) independent experiments with 2‐3 mice per group or (B) two independent experiments (n = 5‐6) with 2‐3 mice per group. *p < 0.05, unpaired t‐test with Welch correction. Supporting Information Fig. 3: WT mice were infected with 2x10^6^ pfu LCMV Docile. (A) Survival and body weight (BW) were monitored for the indicated time period. (B) Viral titers were determined in the indicated organs at day 21 p.i. Symbols represent data from individual mice; horizontal lines indicate means, dashed lines indicate the detection limit. Pooled data (n = 6) from three independent experiments with 1‐3 mice per experiment. Supporting Information Fig. 4: (A, B) WT and MD4 BCR‐transgenic mice specific for hen egg lysozyme (HEL) (Hartley et al., Nature 1991, 353:765‐9) were infected with 200 pfu LCMV Docile. (A) Survival and body weight (BW) were monitored for the indicated time period. (B) Viral titers were determined in the indicated organs at day 9 p.i. Symbols represent data from individual mice; horizontal lines indicate means, dashed lines indicate the detection limit. Data shown are pooled from (A) one (WT, n = 4) or two (MD4, n = 6) independent experiments with 3‐4 mice per experiment or (B) 5‐6 independent experiments (n = 12‐13) with 2‐3 mice per group. *p < 0.05 [file EJI-49-782-s001.pdf]

European Journal of Immunology

Supporting Information

for

DOI 10.1002/eji.201847962

Tobias Straub and Hanspeter Pircher

Enhancing immunity prevents virus-induced T-cell-mediated immunopathology in B cell-deficient mice

© 2019 The Authors. *European Journal of Immunology* published by WILEY-VCH Verlag GmbH & Co. KGaA, Weinheim.

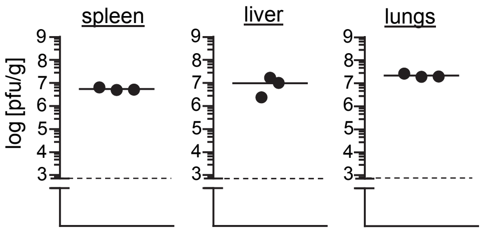

Supporting Information Fig. 1: $CD8^+$ T cell-depleted $B^{-/-}$ mice were infected with 200 pfu LCMV Docile. At day 21 p.i. viral titers in spleen, liver and lungs were determined by focus-forming assay. Symbols represent data from individual mice; data from one experiment are shown (n = 3). The horizontal lines indicate the means, the dashed lines indicate the detection limit.

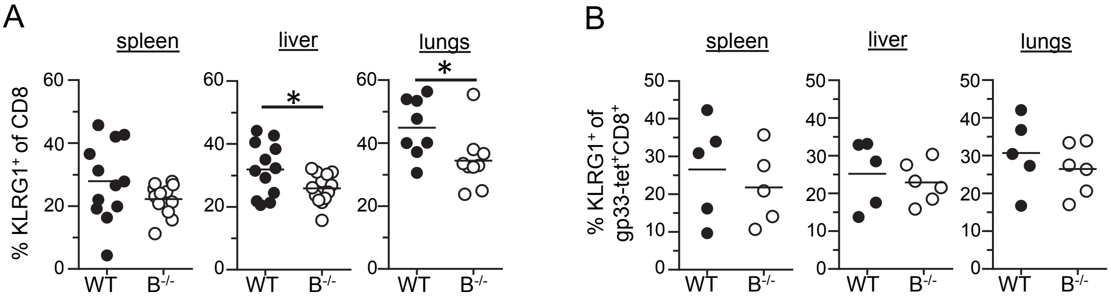

Supporting Information Fig. 2: WT and $B^{-/-}$ mice were infected with 200 pfu LCMV Docile. At day 9 p.i. (A) percentages of $KLRG1^+$ cells among $CD8^+$ T cell and (B) percentages of $KLRG1^+$ cells of gp33-tet$^+$$CD8^+$ T cells were determined. Symbols represent data from individual mice; data shown are pooled from (A) five (spleen, liver; n = 12) or three (lung; n = 8-9) independent experiments with 2-3 mice per group or (B) two independent experiments (n = 5-6) with 2-3 mice per group. * $p < 0.05$, unpaired t-test with Welch correction.

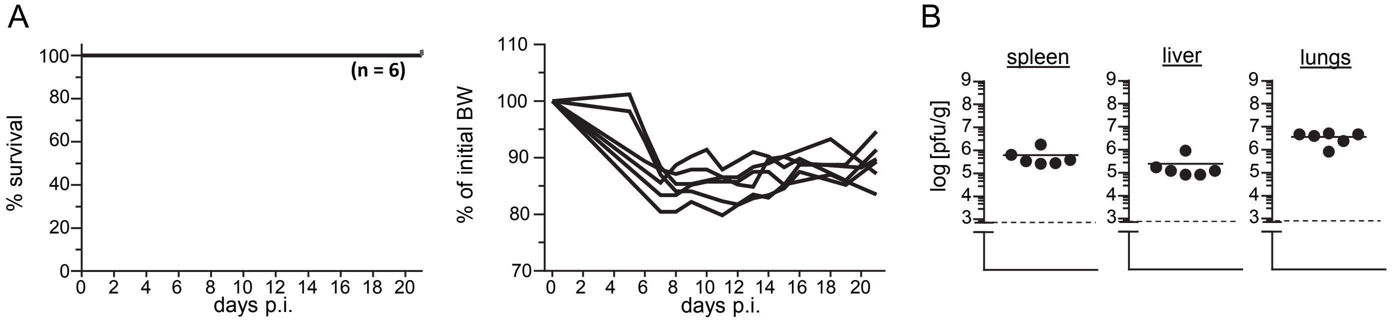

Supporting Information Fig. 3: WT mice were infected with $2x10^6$ pfu LCMV Docile. (A) Survival and body weight (BW) were monitored for the indicated time period. (B) Viral titers were determined in the indicated organs at day 21 p.i.. Symbols represent data from individual mice; horizontal lines indicate means, dashed lines indicate the detection limit. Pooled data (n = 6) from three independent experiments with 1-3 mice per experiment.

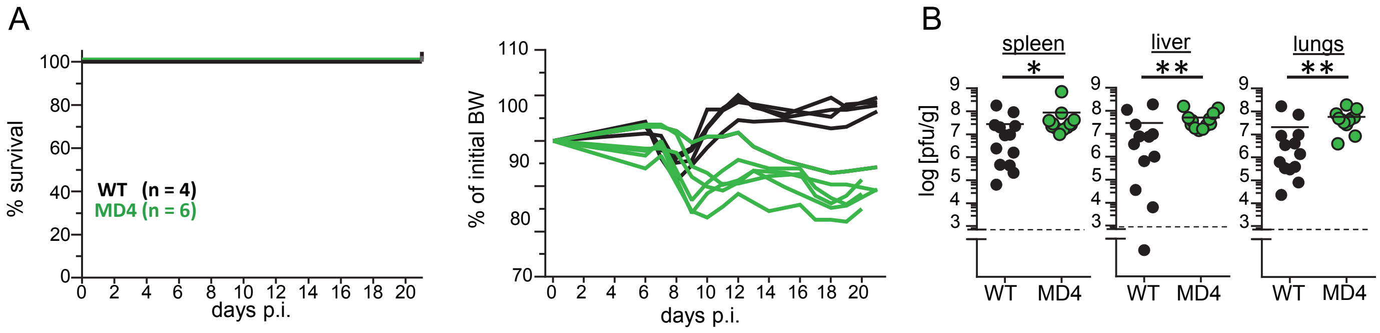

Supporting Information Fig. 4: (A, B) WT and MD4 BCR-transgenic mice specific for hen egg lysozyme (HEL) (Hartley et al., Nature 1991, 353:765-9) were infected with 200 pfu LCMV Docile. (A) Survival and body weight (BW) were monitored for the indicated time period. (B) Viral titers were determined in the indicated organs at day 9 p.i.. Symbols represent data from individual mice; horizontal lines indicate means, dashed lines indicate the detection limit. Data shown are pooled from (A) one (WT, n = 4) or two (MD4, n = 6) independent experiments with 3-4 mice per experiment or (B) 5-6 independent experiments (n = 12-13) with 2-3 mice per group. * $p < 0.05$, ** $p < 0.01$; Mann-Whitney test.

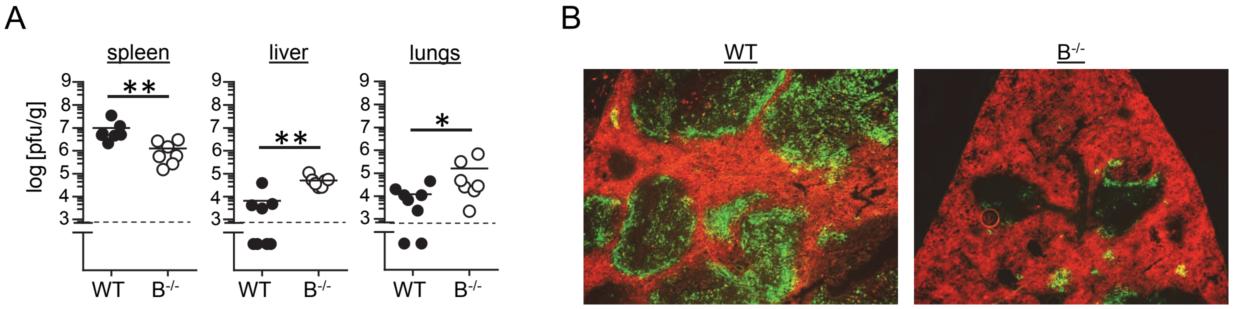

Supporting Information Fig. 5: $B^{-/-}$ mice were infected with 200 pfu LCMV Docile. At day 4 p.i. (A) viral titers in spleen, liver and lungs were determined. Symbols represent data from individual mice; horizontal lines indicate means, dashed lines indicate the detection limit. Data are pooled from 2-3 independent experiments (n = 7) with 2-4 mice per group; (B) splenic sections were stained with rabbit anti-LCMV immune serum and biotinylated anti-F4/80 mAb (clone A3-1, AbD Serotec), followed by streptavidin-AlexaFluor555 (red) and AlexaFluor488-labeled goat anti-rabbit IgG (green, both invitrogen). Exemplary images of two independent experiments are shown. * $p < 0.05$, ** $p < 0.01$, Mann-Whitney test.

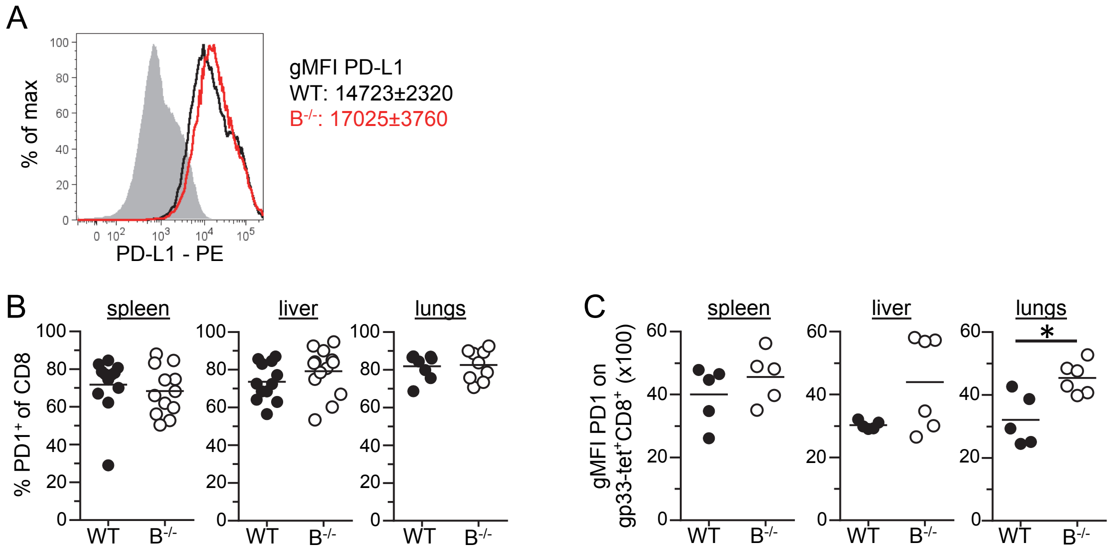

Supporting Information Fig. 6: WT and $B^{-/-}$ mice were infected with 200 pfu LCMV Docile. At day 9 p.i. (A) expression level (geometric mean fluorescence intensity, gMFI) of PD-L1 on $CD45^-CD31^+$ lung endothelial cells and (B) frequency of $PD1^+$ of $CD8^+$ T cells as well as (C) the expression level (gMFI) of PD1 on gp33-tet^+CD8^+ T cells was determined. (A) A representative histogram of one experiment with 3 mice per group (black: WT; red: $B^{-/-}$; grey: uninfected control; numbers to the right indicate gMFI ± s.d.) and (B, C) pooled data from (B) five (spleen, liver; n = 12) or three (lung; n = 8-9) independent experiments with 2-3 mice per group or (C) two independent experiments (n = 5-6) with 2-3 mice per group are shown. Symbols represent data from individual mice, horizontal bars indicate the means. * $p < 0.05$, unpaired t-test with Welch correction.

.

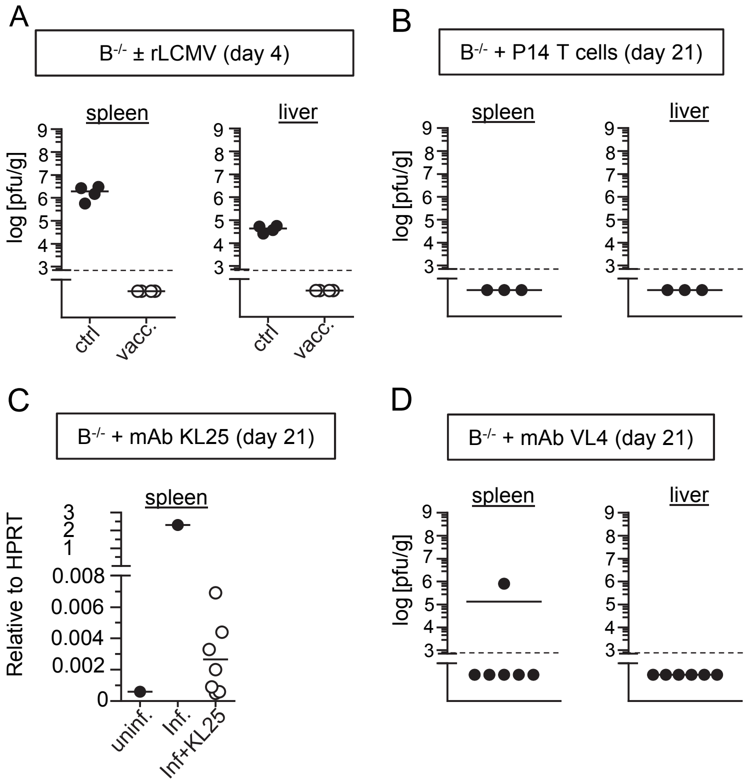

Supporting Information Fig. 7: (A) $B^{-/-}$ mice were vaccinated (vacc.) with replication-deficient rLCMV/WEGPΔGlc6,9 (8×10^4 pfu) three weeks prior to challenge with 200 pfu LCMV Docile. Non-vaccinated $B^{-/-}$ mice were included as a control (ctrl). Viral titers were determined by focus-forming assay at day 4 p.i.. (B) $B^{-/-}$ mice were adoptively transferred (i.v.) with 5×10^5 P14 TCR tg $CD8^+$ T cells at the day of infection with 200 pfu LCMV Docile. Viral titers were determined at day 21 p.i.. (C) LCMV Docile-infected $B^{-/-}$ mice were treated once with neutralizing LCMV GP-specific mAb KL25 (1 mg) at day 4 p.i.. At day 21, viral load was determined by qPCR. (D) LCMV Docile-infected $B^{-/-}$ mice were treated once with non-neutralizing LCMV NP-specific mAb VL4 (0.5 mg) at day 1 p.i.. Viral titers were determined at day 21 p.i. using focus-forming assay. (A-D) Symbols represent values from individual mice, horizontal lines indicate means, dashed lines indicate the detection limit; data are pooled from two to three independent experiments; uninfected and infected controls in (C) are derived from one experiment (n = 1).

LCMV qPCR was performed as follows: RNA was prepared from splenic tissue using phenol (TriReagent, Sigma)-chloroform-extraction followed by EtOH-sodium acetate-precipitation. cDNA was prepared using the High Capacity cDNA Reverse Transcription Kit (Applied Biosystems) and qPCR was performed using the KAPA SYBR FAST qPCR Master Mix Kit (Kappa Biosystems) and the following primers:
LCMV Docile NP fw 5'-CCTCACTGTTGCTCGGCTTA-3',
LCMV Docile NP rev 5'-ACAGCAGTCCAGCATCAACA-3',
mouse HPRT fw 5'-GTTAAGCAGTACAGCCCCAAA-3',
mouse HPRT rev 5'-AGGGCATATCCAACAACAAACTT-3'.
PCR conditions: 50°C 2 min.; 95°C 10 min.; 40 cycles of 95°C 15 sec.; 60°C 20 sec.. Samples were run in triplicates on a QuantStudio 5 real-time PCR system and analyzed with QuantStudio Design&Analysis software (both Thermo Fisher Scientific) .

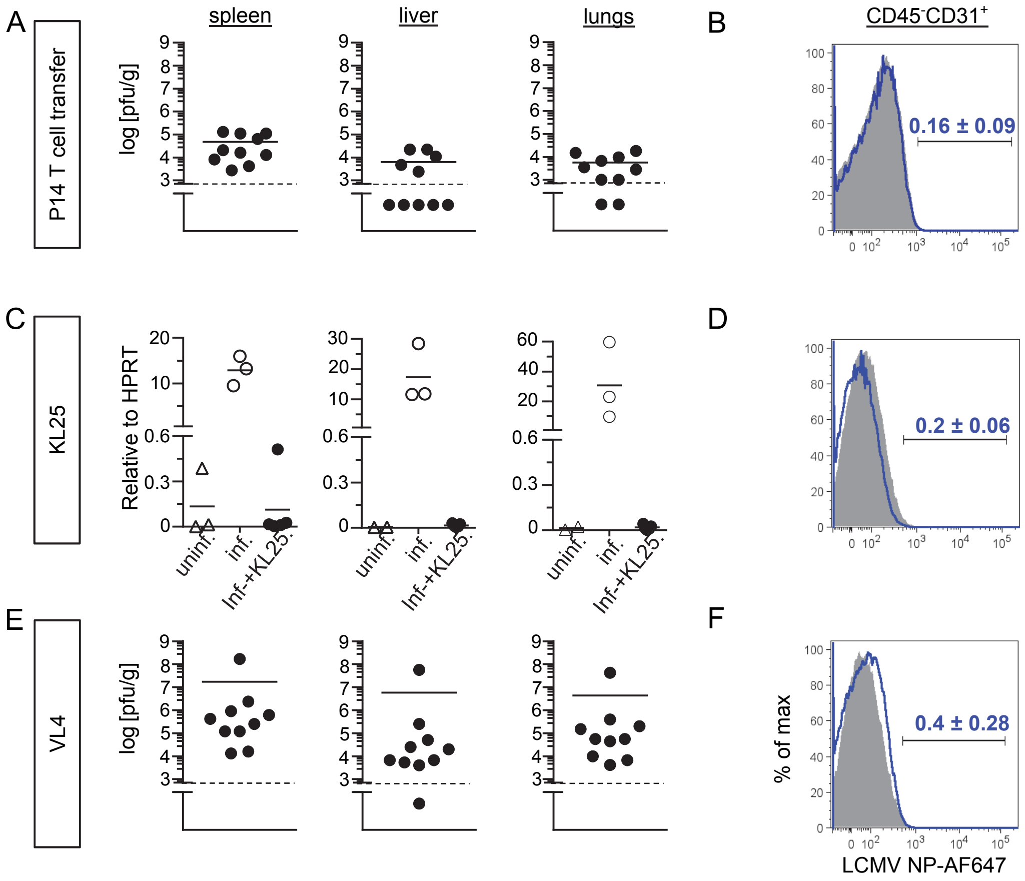

Supporting Information Fig. 8: $B^{-/-}$ mice were infected with 200 pfu LCMV Docile and treated with (A) $5x10^5$ P14 T cells (day 0 p.i.), (B) 1 mg mAb KL25 (day 4 p.i.) or (C) 500 µg mAb VL4 (day 1 p.i.). At day 9 p.i. (A, C, E) viral load in spleen, liver and lungs and (B, D, F) frequency of LCMV NP^+ lung endothelial cells ($CD45^-CD31^+$) were determined. Pooled data and representative histograms from three (A, C, E) or two (B, D, F) independent experiments are shown (n = 8-10 or n = 5-6, respectively) with 2-4 mice per experiment. Data for infected and uninfected controls in (C) are derived from one experiment (n = 3). Viral loads from untreated controls are shown in Fig. 2A. (A, C, E) Symbols represent data from individual mice; horizontal lines indicate means, dashed lines indicate detection limit. (C, D, F) Numbers in histograms indicate percentage of LCMV NP^+ cell ± s.d..

A

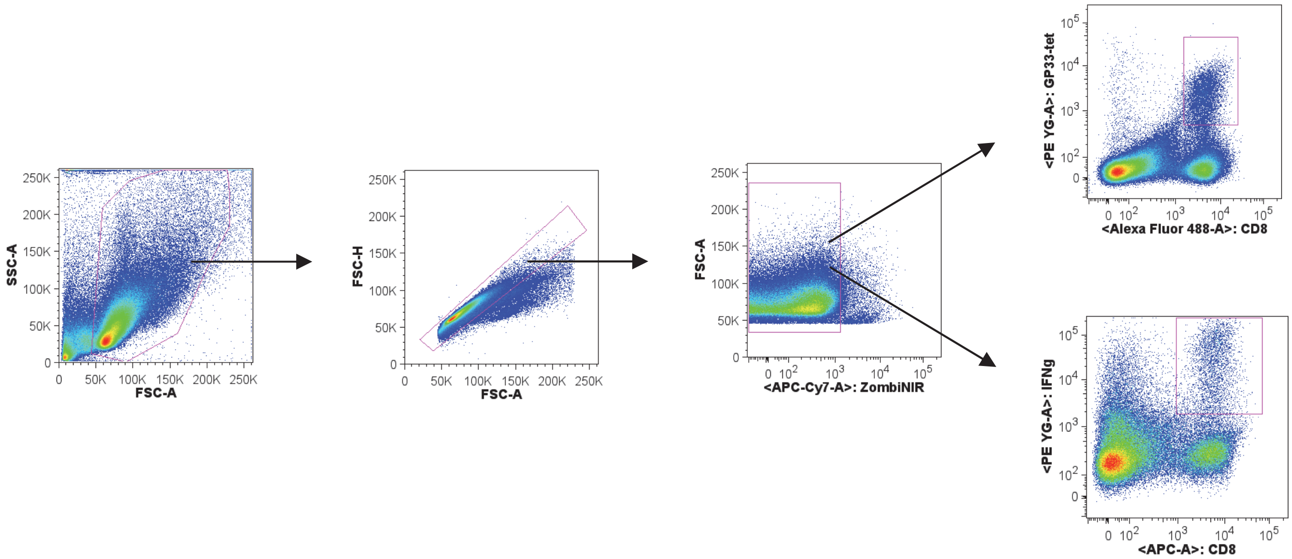

B

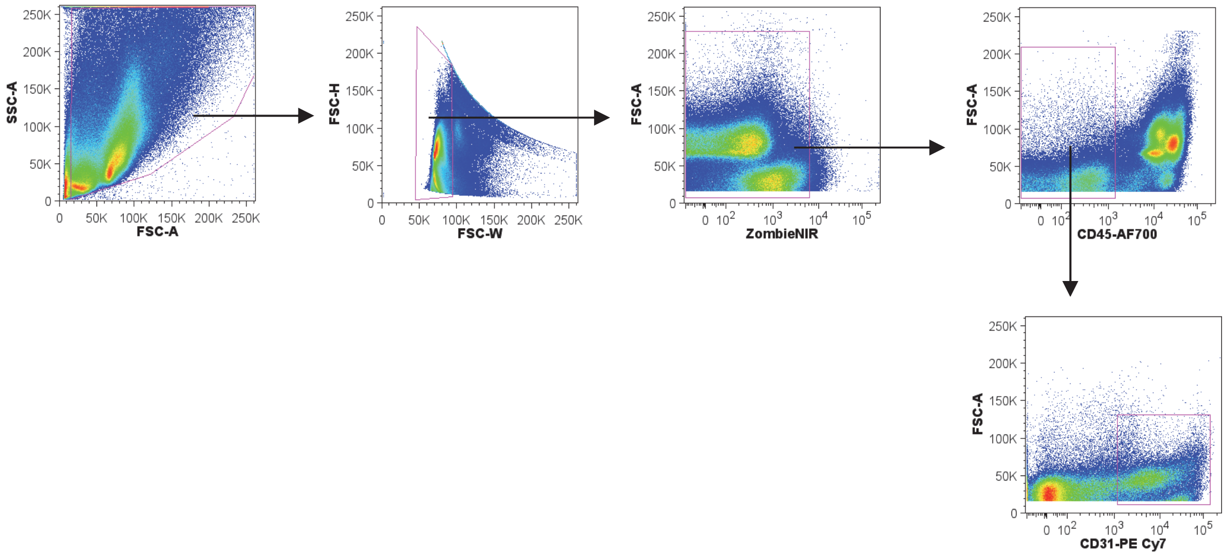

Supporting Information Fig. 9:
(A) Gating strategy used in Figure 1C
(B) Gating Strategy used in Figure 2D and E
